# Genetic correlation between female infertility and mental health and lifestyle factors: A linkage disequilibrium score regression study

**DOI:** 10.1002/hsr2.797

**Published:** 2022-09-01

**Authors:** Miao Ma, Lu Guo, Xiaocheng Liu, Yingxin Zheng, Chao Gu, Bin Li

**Affiliations:** ^1^ Department of Gynecology and Obstetrics Obstetrics and Gynecology Hospital of Fudan University Shanghai China

**Keywords:** female, genetic correlation, infertility, lifestyle, mental health

## Abstract

**Background and Aims:**

Female fertility is a heterogeneous condition of complete psychosocial and physical well‐being. Observational studies have revealed that women with infertility have varying degrees of poor mental status and lifestyle choices in varying degrees. However, the genetic contribution to female infertility remains elusive. Our study aimed to explore the genetic correlations between female infertility and mental health and lifestyle factors.

**Methods:**

The genome‐wide association study (GWAS) data sets of characteristics related to mental health and lifestyle were obtained from the IEU OpenGWAS database. The GWAS data sets of female infertility were derived from the Finggen database. Linkage disequilibrium score regression was performed to systematically estimate the pairwise genetic correlations between female infertility and a set of mental health‐ and lifestyle‐related traits.

**Results:**

The genetic correlation analysis revealed a moderate and positive genetic correlation between depressive symptoms, major depressive disorder, and female infertility. Similarly, worry and the personality trait of neuroticism displayed a moderate positive genetic correlation with female infertility. Adversely, a negative and moderate genetic correlation was observed between strenuous sports or exercises and female infertility.

**Conclusion:**

The study demonstrated genetic correlations between female infertility and mental health status, including depression, worry, and neuroticism. Additionally, we observed that females with better physical activity may have reduced risks of female infertility. These findings would serve as a fundamental resource for understanding the genetic mechanisms of the effects of mental health and lifestyle factors on female infertility.

## 1 INTRODUCTION

Infertility is defined as failure to achieve pregnancy within 12 months of unprotected intercourse or therapeutic donor insemination in females <35 years or within 6 months of that in females >35 years.^1^ With delayed child‐bearing age, there has been an increasing infertility rate globally. Infertility affects 10%–25% of couples of fertility age (18–45 years of age) worldwide.^2^, ^3^, ^4^ It is a couples' issue; however, the existing treatments for infertility are mainly for women.^3^, ^5^ The common reasons for female infertility include tubal obstruction, ovulatory function, and structural abnormalities.^1^ The risk factors may impact female infertility variably. The risk factors include age, reduced ovarian reserve, ovulatory dysfunction, menstrual disorders, infertility for >3 years, and endometriosis.^6^ However, for females, infertility may have ramifications beyond reproductive health. Female fertility is a state of complete psychosocial and physical well‐being. Attention has been drawn to the association between female infertility and mental health disorders and lifestyle.^7^ For instance, observational studies have demonstrated that females with infertility have varying degrees of anxiety and depression.^8^, ^9^, ^10^ Additionally, infertility is a significant psychological stressor, which may be associated with marital conflict and domestic violence.^11^ Poor mental health conditions including depression aggravate infertility. Moreover, a recent review has revealed an association between multiple lifestyle factors, such as weight and exercise, and fertility.^12^ Female infertility is a heterogeneous condition, the genetic contribution to which remains elusive.^13^, ^14^, ^15^ Genetic correlation, which is the proportion of variance shared by two or more traits owing to common genetic causes, can explain the causes underlying complex diseases and traits.^16^ Genome‐wide association studies (GWAS), based on a large sample size with an array of data, are a tool for detecting genetic variations associated with the target diseases or traits across the whole genome.^17^ Linkage disequilibrium score regression (LDSC), a technique that only requires GWAS summary statistics, has become a popular approach to estimating the genetic correlation of infertility.^18^ A better understanding of the genetic correlation between infertility and other diseases or traits would provide new insights into the etiology of infertility and enable physicians to personalize counseling for females with infertility

In this study, we aimed to evaluate the common genetic components of female infertility and other diseases or traits. We used cross‐trait LDSC to systematically estimate the pairwise genetic correlation of female infertility with mental health and lifestyle factors.

## 2 MATERIALS AND METHODS

### 2.1 Study sample and data sources

#### 2.1.1 Female infertility

The FinnGen study, an ongoing nationwide cohort study launched in 2017, combines genetic data from the Finnish Biobanks with health record data from the Finnish health registries. In FinnGen, GWAS summary statistics for >1800 phenotypes/endpoints have been publicly released. Finland is a best‐studied genetic isolate and a unique gene pool of the Finns. GWAS summary‐level data (*β*‐coefficients and corresponding standard errors) from FinnGen on female infertility comprised 6481 cases and 68,969 controls (https://r5.finngen.fi/pheno/N14_FEMALEINFERT). The inclusion criterion was females diagnosed with infertility. The diagnosis of female infertility in FinnGen was defined by N97 in the International Classification of Diseases (ICD) 10 code. The exclusion criterion was male infertility (the inability of the male to fertilize an ovum after a certain period of unprotected intercourse). Females who were not diagnosed with infertility were considered controls. Methods (participating cohorts, data collection, genotyping, and data analysis) are demonstrated on the FinnGen webpage.

#### 2.1.2 Mental health‐related traits

The GWAS summary statistics of mental health‐ and lifestyle‐related traits were selected from the IEU OpenGWAS database (https://gwas.mrcieu.ac.uk). This website provides open access to over 10,000 complete GWAS summary data sets.^18^ For multiple GWAS data sets available for a single trait, only the largest study was used. Cases of major depressive disorder,^19^ anorexia nervosa,^20^ schizophrenia,^21^ autism spectrum disorder,^22^ and neuroticism^23^ were defined by the ICD codes or the Diagnostic and Statistical Manual of Mental Disorders criteria (Table 1). The trait linked to depressive symptoms was operationalized by combining responses to a series of questions (i.e., the frequency with which the respondent experienced feelings of unenthusiasm/disinterest and depression/hopelessness in the previous 2 weeks).^24^ Similarly, worry^25^ was evaluated via questions (i.e., “Are you worried?,” “Do you suffer from nervousness?,” “Would you call yourself a nervous person?,” and “Would you describe yourself as tense or highly strung”). The detailed cohort descriptions and information about genotyping, imputation, and association analysis are available in the published studies. Most mental health condition‐related GWAS utilized the UK Biobank data set, which is a large population‐based study of adults aged 40–69 years residing in England, Scotland, and Wales.^26^ Besides the UK Biobank, some of them were obtained from the Psychiatric Genomics Consortium (PGC), Genetic Epidemiology Research on Aging (GERA), and Genetic Consortium for Anorexia Nervosa (GCAN).

**Table 1:** Mental health‐related traits utilized in genetic correlation analyses

GWAS ID^a^	Traits	GWAS data source	Consortium	Sample size	Number of SNPs
ieu‐a‐805	Major depressive disorder	Ripke et al.^27^	PGC	9240	1,235,110
ieu‐a‐1185	Autism spectrum disorder	Grove et al.^22^	PGC	46,351	9,112,386
ieu‐a‐45	Anorexia nervosa	Boraska et al.^20^	GCAN	2442	1,149,254
ieu‐b‐41	Bipolar disorder	Stahl et al.^28^	PGC	51,710	13,413,244
ieu‐b‐42	Schizophrenia	SWG‐PGC^21^	PGC	77,096	15,358,497
ebi‐a‐GCST006940	Neuroticism	Nagel et al.^23^	UKB	380,506	10,824,976
ebi‐a‐GCST006475	Depressive symptoms	Okbay et al.^24^	UKB GERA PGC	180,866	6,019,632
ebi‐a‐GCST006478	Worry	Nagel et al.^25^	PGC UKB 23andMe	348,219	10,828,862

Abbreviations: GCAN, Genetic Consortium for Anorexia Nervosa; GERA, Genetic Epidemiology Research on Aging; GWAS, genome‐wide association study; PGC, Psychiatric Genomics Consortium; SNPs, single‐nucleotide polymorphisms; SWG‐PGC, Schizophrenia Working Group of the Psychiatric Genomics Consortium; UKB, UK Biobank data set.

^a^ GWAS ID in IEU OpenGWAS database.

#### 2.1.3 Lifestyle factors

The following lifestyle factors were included in our study: Sleep duration, alcoholic consumption, smoking behavior, vitamin D levels, and strenuous sports or exercises (Table 2). Strenuous sports or exercises, such as fast cycling, aerobics, and heavy lifting, induce sweat or heavy breathing.^29^ Lifestyle‐related GWAS utilized the UK Biobank data set, GWAS and Sequencing Consortium of Alcohol and Nicotine use (GSCAN), and SUNLIGHT Consortium. Ethical approval and participant consent for each study contributing to the GWAS are available in the original publications.

**Table 2:** Lifestyle‐related traits utilized in genetic correlation analyses

GWAS ID	Traits	GWAS data source	Consortium	Sample size	Number of SNPs
ieu‐a‐1088	Sleep duration	Jones et al.^30^	UKB	128,266	16,761,226
ebi‐a‐GCST006100	Strenuous sports or exercises	Klimentidis et al.^29^	UKB	350,492	11,807,536
ebi‐a‐GCST005367	Vitamin D levels	Jiang et al.^31^	SUNLIGHT	79,366	2,538,249
ieu‐b‐142	Cigarettes smoked per day	Liu et al.^32^	GSCAN	249,752	12,003,613
ieu‐b‐73	Alcoholic drinks per week	Liu et al.^32^	GSCAN	335,394	11,887,865

Abbreviations: GSCAN, GWAS and Sequencing Consortium of Alcohol and Nicotine use; GWAS, genome‐wide association study; SNP, single‐nucleotide polymorphism;  SUNLIGHT, SUNLIGHT Consortium; UKB, UK Biobank database.

### 2.2 Statistical analysis

LDSC analysis is a powerful tool to investigate the shared genetic components (*r*_g_, genetic correlation) between common traits or diseases based on GWAS summary statistics. Bivariate LDSC analyses were performed using the LDSC software version 1.0.1 (https://github.com/bulik/ldsc).^16^ The LDSC method did not require individual‐level genotype data. GWAS summary statistics were used to regress *χ* ^2^ statistics on their LD scores.^33^ Genetic correlations were calculated using overlapping single‐nucleotide polymorphisms (SNPs) from the GWAS summary statistic files. The SNPs with minor allele frequencies >0.01 were included in LDSC. The Benjamin−Hochberg false discovery rate (FDR) correction was obtained to account for multiple testing, and the FDR‐adjusted *p* < 0.05 was considered significant. All statistical tests were two‐sided. The analyses were performed using R version 3.6.3 statistical software (R Foundation for Statistical Computing; https://www.R-project.org/).

## 3 RESULTS

### 3.1 Genetic correlations between female infertility and mental health disorders

**Figure 1:**
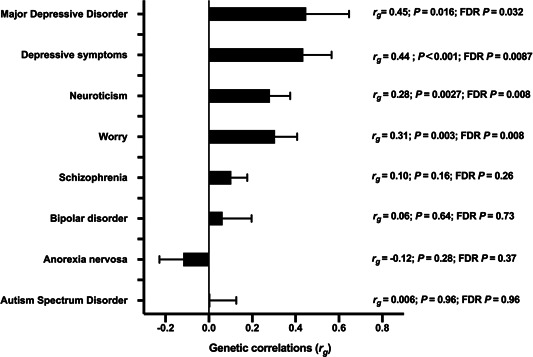
Genetic correlations (*r*_g_) between mental health‐related traits and female infertility. Genetic correlation estimates obtained through linkage disequilibrium score regression are presented; false discovery rate (FDR)‐adjusted *p* value is listed on the right. Error bars represent standard error.

The genetic correlations (*r*_g_) and standard errors between female infertility and traits of mental health status are presented in Figure 1. The genetic correlation analysis revealed a moderate and positive genetic correlation between female infertility and major depressive disorder (*r*_g_ = 0.45, *p* = 0.016, FDR‐adjusted *p* = 0.032). The genetic correlation result of depressive symptoms was similar to that of major depressive disorder. The genetic correlation was moderate and significant (*r*_g_ = 0.44, *p* < 0.001, FDR‐adjusted *p* = 0.0087). The personality trait of neuroticism revealed moderate positive genetic correlation with female infertility (*r*_g_ = 0.28, *p* =  0.0027, FDR‐adjusted *p* = 0.008). Additionally, moderate genetic correlation was observed between female infertility and worry (*r*_g_ = 0.31, *p* =  0.003, FDR‐adjusted *p* = 0.008).

The genetic correlations between female infertility and other traits of mental health conditions, including anorexia nervosa, bipolar disorder, schizophrenia, and autism spectrum disorder, *r*_g_, were −0.12 to 0.10, all of which were statistically insignificant (*p*  > 0.05).

### 3.2 Genetic correlations between female infertility and lifestyle factors

**Figure 2:**
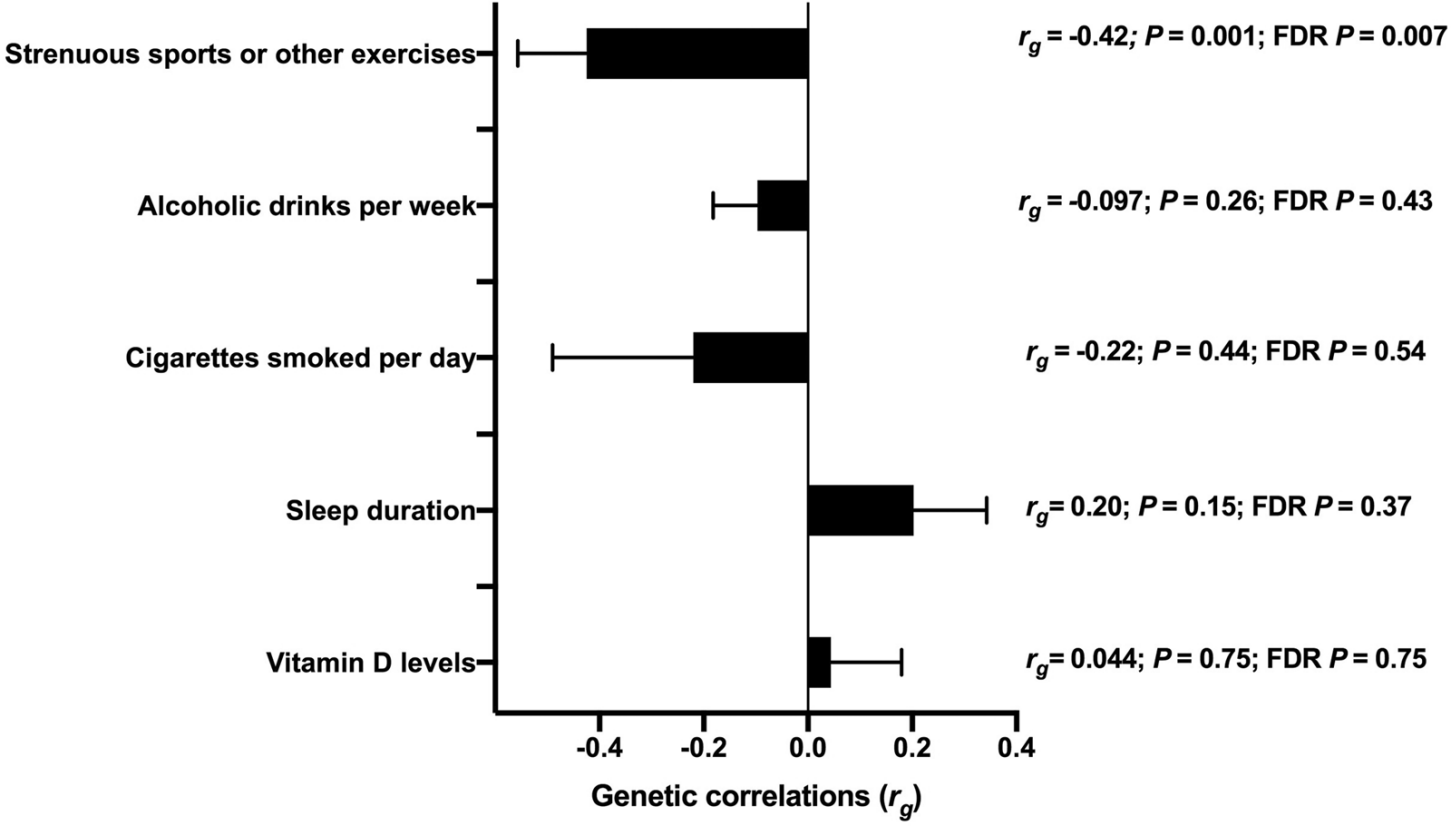
Genetic correlations (*r*_g_) between lifestyle‐related traits and female infertility. Genetic correlation estimates obtained through linkage disequilibrium score regression are presented; false discovery rate (FDR)‐adjusted *p* value is listed on the right. Error bars represent standard error.

The genetic correlations between female infertility and lifestyle factors are presented in Figure 2. A negative and moderate genetic correlation was observed between strenuous sports or exercises and female infertility (*r*_g_  =  −0.42, *p*  =  0.001, FDR‐adjusted *p* = 0.007). We observed no genetic correlation between female infertility and sleep duration (*r*_g_ =  0.20, FDR‐adjusted *p* = 0.37) and smoking (*r*_g_  = −0.22, FDR‐adjusted *p* = 0.54). Similarly, we observed no genetic correlation between alcohol, vitamin D levels, and female infertility (*r*_g_ = −0.097 and 0.044, respectively; *p*  > 0.05).

## 4 DISCUSSION

To our knowledge, our study is the first to investigate genetic correlations between female infertility and multiple mental health‐related traits and lifestyle factors. We demonstrated significant genetic correlations between female infertility and poor mental health and strenuous sports

Previous studies have demonstrated that women with infertility reported elevated levels of anxiety and depression.^8^, ^9^, ^10^ In this study, female infertility revealed significant shared genetic components with a range of mental health‐related traits, consistent with previous epidemiological reports. A different interpretation of the effect of depressive symptoms and major depressive disorder on female infertility is required owing to the genetic overlap between depression and female infertility. The SNPs that predispose to depression also increase the risk for depression and major depressive disorder, which should be considered in addition to female infertility. This study supports previous studies displaying that depression is associated with increased reporting and sensitivity to female infertility. The genetic correlation between worry and female infertility observed in our study demonstrated that anxiety increases the risk for infertility and displayed the genetic influences on anxiety and female infertility.

We observed a significant genetic correlation between neuroticism and female infertility, which has been reported in previous observational studies.^34^, ^35^ Females via personality traits, such as neuroticism, prime them to respond negatively to fertility. These results indicated that there is some genetic overlap between neuroticism and female infertility at the molecular level. It has been reported that the genetic signal of neuroticism partly originates from two genetically distinguishable subclusters (“depressed affect” and “worry”).^25^ We examined genetic correlations between depressive symptoms, worry, and female infertility. We observed depression to be more strongly associated with female infertility than worry. This further suggested that the genetic association may be stronger between female infertility and depression than between female infertility and worry.

We observed a modest and significant genetic correlation between strenuous exercise and female infertility using the LDSC approach. The negative correlation reflected a difference in genetic background between female infertility and strenuous exercises. Physically active females were more likely to be fertile, consistent with a previous observational study reporting that more females with normal fertility engaged in moderate and vigorous activities.^36^ However, other conservative studies have revealed that among females, excessive exercise may lead to hypothalamic amenorrhea, causing short‐term infertility.^37^ Generally, levels of engagement in physical activity vary across individuals. Recall, comprehension, and social desirability bias are prevalent in the measurement of physical activity, whereas phenotypic agreement in strenuous and excessive exercises is generally poor between subjective and objective measures.^38^ Interindividual variation is likely to exist in relevant studies. Regardless, our data did not provide any insight into the impact of light or moderate physical activity on infertility.

Smoking phenotypes and alcohol use are genetically correlated with many health conditions.^39^ In our study, we did not detect a significant correlation between alcohol use and smoking and female infertility. Evidence has revealed a significant association between smoking and reduced female fertility.^39^ However, the impact of alcohol use on female fertility is inconsistent. Some studies have revealed that moderate alcohol use may be unrelated to female fertility albeit increased the risk of adverse pregnancy outcomes.^39^, ^40^ Although there was no genetic overlap between smoking, alcohol use, and female infertility, the adverse effects of smoking and alcohol use should be further evaluated.

There were several strengths to our study. First, our study addressed the genetic correlation between female infertility and other diseases or traits. Second, we believe that LDSC is the most effective tool for genetic correlation analysis since the summary statistics are available for much larger sample sizes than those with individual genotype data. Third, in contrast to observational studies, our LDSC analysis supported a reliable methodology to assess the association between complex traits while minimizing the possibility of bias owing to unknown confounding. This study had some limitations. We did not estimate sex‐based genetic correlation. Female infertility phenotypes only included females, whereas samples of mental health disorders and lifestyle‐related factors included both sexes. Furthermore, most phenotypes used the UK Biobank data, which limits the generalizability of results to other ancestries. For a larger sample size, large‐scale genetic studies that replicate findings across other ancestry groups will be useful. Finally, the clinical significance of some poor or modest genetic correlations remained elusive. Future work with an increased sample size to replicate these findings is warranted.

## 5 CONCLUSION

Conclusively, by utilizing the LDSC approach, we evaluated the association between mental health status, lifestyle factors, and female infertility. Our study identified significant and positive genetic correlations between multiple mental health conditions and female infertility. We also revealed that strenuous exercise is negatively correlated with female infertility. We believe that our findings would serve as a fundamental resource for understanding the genetic mechanisms of the effects of depression, anxiety, neuroticism, and lifestyle factors on female infertility. Furthermore, we believe that our study lends support to future research into the mental health and lifestyle of females with infertility.

## AUTHOR CONTRIBUTIONS

*Conceptualization*: Miao Ma, Lu Guo, and Bin Li. *Formal analysis*: Miao Ma, Xiaocheng Liu, and Yingxin Zheng. *Writing—review and editing*: Chao Gu and Bin Li. *Writing—original draft*: Miao Ma. All authors have read and approved the final version of the manuscript. Miao Ma had complete access to all the data in this study and takes complete responsibility for the integrity of the data and the accuracy of the data analysis.

## CONFLICT OF INTEREST

The authors declare no conflict of interest.

## TRANSPARENCY STATEMENT

Miao Ma affirms that this manuscript is an honest, accurate, and transparent account of the study that has been reported, that no important aspects of the study have been omitted, and that any discrepancies from the study as planned (and, if relevant, registered) have been explained.

## Data Availability

The data supporting the findings of this study are available on the FinnGen database (https://r5.finngen.fi/pheno/N14_FEMALEINFERT) and IEU OpenGWAS database (https://gwas.mrcieu.ac.uk).
